# Social and Ethical Implications of Digital Crisis Technologies: Case Study of Pandemic Simulation Models During the COVID-19 Pandemic

**DOI:** 10.2196/45723

**Published:** 2024-01-16

**Authors:** Gabriel Bartl

**Affiliations:** 1 Centre Marc Bloch Berlin Germany

**Keywords:** public health technologies, simulation models, algorithmic governance, preparedness, crisis, uncertainty, and ignorance, social implications of mathematical modeling, normativity, transparency, legitimacy

## Abstract

**Background:**

Responses to public health crises are increasingly technological in nature, as the prominence of COVID-19–related statistics and simulations amply demonstrates. However, the use of technologies is preconditional and has various implications. These implications can not only affect acceptance but also challenge the acceptability of these technologies with regard to the ethical and normative dimension.

**Objective:**

This study focuses on pandemic simulation models as algorithmic governance tools that played a central role in political decision-making during the COVID-19 pandemic. To assess the social implications of pandemic simulation models, the premises of data collection, sorting, and evaluation must be disclosed and reflected upon. Consequently, the social construction principles of digital health technologies must be revealed and examined for their effects with regard to social, ethical, and ultimately political issues.

**Methods:**

This case study starts with a systematization of different simulation approaches to create a typology of pandemic simulation models. On the basis of this, various properties, functions, and challenges of these simulation models are revealed and discussed in detail from a socioscientific point of view.

**Results:**

The typology of pandemic simulation methods reveals the diversity of model-driven handling of pandemic threats. However, it is reasonable to assume that the use of simulation models could increasingly shift toward agent-based or artificial intelligence models in the future, thus promoting the logic of algorithmic decision-making in response to public health crises. As algorithmic decision-making focuses more on predicting future dynamics than statistical practices of assessing pandemic events, this study discusses this development in detail, resulting in an operationalized overview of the key social and ethical issues related to pandemic crisis technologies.

**Conclusions:**

This study identifies 3 major recommendations for the future of pandemic crisis technologies.

## Introduction

### Knowledge Production and the Political Significance of Data in Times of Uncertainty

The COVID-19 pandemic highlighted 2 opposing trends that have been lost in the public debate. On the one hand, the pandemic showed that “digital prediction tools increasingly complement or replace other practices of coping with an uncertain future” [1]. In contrast, the pandemic blatantly illustrated the complicated relationship between scientific knowledge and ignorance in the context of political decisions and social dynamics in dealing with crises. In accordance with the Bauman [2] diagnosis of the “end of unambiguousness,” Habermas [3] stated that “There has never been so much knowledge about our not-knowing and about the compulsion to act and live under uncertainty.”

These 2 opposing trends point to the observation that knowledge is ambivalent, increasingly fragile, and ambiguous but simultaneously acts as a central resource [4]. This has not least to do with the fact that there is no such thing as a single, definitive science that produces objective findings. In the context of digital health technologies, this seems to be a paradox as the increasing use and implementation contrasts with the possibility of clear evidence production. The growing demand for scientific expertise in times of crisis and the simultaneous lack of clarity in scientific knowledge represent a paradox that cannot be resolved and seems to be becoming increasingly severe [5].

### The Ideology of “Technological Fix” in Public Health Crisis Management

Especially during the COVID-19 pandemic, it has been shown that promises of unambiguity and evidence must be illusory and that both ascriptions must be contrasted with the hypothesis of a “situatedness of knowledge” [6]. Thus, knowledge must be reflected in its contexts of production. This means that a simple translation of the relationship between knowledge and ignorance into the political sphere, for example, within preventive health programs, must be the subject of debate while including diverse stakeholders such as health experts, policy makers, and “lay persons” [7]. Finally, this also indicates that we must speak of science in the plural and that policy cannot claim to follow a single, definitive science when there are conflicting findings that compete with each other.

### Objective

This situation has multifaceted consequences and implications for knowledge-based crisis technologies in public health. Although temporal pressure and threat under conditions of uncertainty and insecurity are elementary characteristics of crises [8], multiple crises, such as global pandemics, are characterized by the fact that these factors interact even more intensively not least because the level of interactions, which are difficult to anticipate, increases the degree of complexity, uncertainty, and ignorance. Those experiences with limited control capabilities have paved the way for a security strategy of preparedness that has prevailed especially in the field of global public health where gaining importance of the “precautionary principle” can be observed. In the same breath, different modes of resilience gained attraction, especially with regard to the emergence of preparedness practices and strategies that are often technological in nature [9]. The rise of a solutionist ideology of “technological fix” [10] in public health crisis management is expressed especially in the use of computer models that simulate the spread of the coronavirus. As certain simulation techniques are likely to play an even more significant role in the pandemic technologies of the future, the following sections will take a deeper look into the premises, logics, and differences of simulation models before shedding light on the social, ethical, and political implications of digital crisis technologies in the context of public health.

Undoubtedly, computer simulations grounded on epidemiological models have played a crucial role in handling the pandemic. This happened, for example, because these models provided orientation knowledge in a crisis situation with considerable temporal pressure. Therefore, it could be argued that one of the central attributions to the use of monitoring technologies during crises such as pandemics is the expected time gains that are essential to preserve the decision makers’ ability to act.

## Methods

### Overview

Methodologically, this case study is based on a systematization of different simulation approaches to create a typology of pandemic simulation models. On the basis of this, various properties, functions, and challenges of these simulation models are revealed, such as their perception as visual representations or certain problems in converting complexity into numerical parameters. Subsequently, to what extent pandemic simulation models can be considered as algorithmic governance tools is explored. Thus, the methodological approach is closely interwoven with a discussion of the ethical, social, and political implications of using simulations.

### Ethical Considerations

No ethics approval was requested, as the methodological approach is based on a description and comparison of pandemic simulation models. Therefore, no personal data were collected, and only relevant literature was referenced to clarify the functioning of the corresponding simulation models.

## Results

Concerning their functional logics, simulation models are to be distinguished from other forms of crisis governance, such as, for example, early warning systems that aim to forecast the future by making use of prognostic methods. In contrast, the vast majority of simulation models are scenario-based approaches that are not grounded on probabilistic calculations but contain different ways of dealing with uncertainty and crises by comparing different courses of action and by considering both the effects of the assumed political countermeasures and the respective societal coping modes. Simulations thus provide policy makers with information by contrasting measures with their possible effects within algorithmic procedures.

The interventions of the policy makers are thus tested according to a *what if* logic so that they can be compared in terms of their effects.

In this respect, epidemiological computer simulations on the one side can be seen as “technologies of preparedness” [11] that stand in contrast to purely prognostic approaches aiming at mitigating future threats by number-based calculations. On the other side, specific future-oriented containment strategies can also be simulated during a public health crisis that has already occurred and is still ongoing, for example, to identify bottlenecks in the infrastructure. What both have in common is a doubling of reality [12]. They are designed to ensure consistency, speed, efficiency, and removal of human bias and error. Hälterlein [12] pointed out that within computer models, different countermeasures together with their uncertain outcomes are simulated based on algorithmic processes. This led him to argue that the use of epidemiological computer simulations is to be understood as a process of algorithmic decision-making [12].

There are a number of different simulation models that are briefly presented and distinguished from each other in the following sections. However, Table 1 represents an ideal typical listing of pandemic model simulations, which means that clear distinctions often cannot be made and single modeling techniques may also partially overlap. It is also noticeable that, although models are always accompanied by a certain reduction in complexity, this parameter can vary dramatically with respect to specific simulation techniques. In addition, the claims of the models vary, as some of them have prognostic ambitions to some degree. Finally, it should be mentioned that the relevance of the single models to policy making varied from country to country during the pandemic [13].

**Table 1:** Typology of COVID-19 pandemic simulation models.

Modeling techniques	Specifications	Features
Compartmental models	Division of the population into different groups, for example, SEIR^a^	Infection dynamics are modeled with respect to the transitions between those groups
Statistical models	Development and testing of theories through causal explanation, prediction, and description (eg, growth models or time series)	Explanatory power of models corresponds with predictive power
Bayesian methods	Specific statistical approach: available knowledge about statistical parameters is merged with data from observed information	Bayesian methods can be used even with a small data base
Network models	Analysis of the distributions in the network links to be able to distinguish certain network types from each other	Search for patterns in the contact structures
Agent-based models	The population to be modeled is divided into subgroups and is grounded on agents with different individual behaviors	Social context is central in contrast to other modeling techniques
AI^b^ models	(Deep) learning algorithms, neural networks, or adaptive agents adjusting their behaviors to changing environmental conditions	Aiming more on prediction (eg, incidence rates) and forecasting than on description
Hybrid models	Combination of different modeling techniques	Depending on the modeling techniques that are combined (eg, SEIR with machine learning to predict the evolution of the pandemic [13])

^a^SEIR: susceptible, exposed, infectious, recovered.

^b^AI: artificial intelligence.

The most common and also the most popular simulation models during the pandemic were the so-called compartmental models. Here, the susceptible, infectious, recovered (SIR) [14] models can be distinguished from the susceptible, exposed, infectious, recovered (SEIR) models, although many more variants exist. For example, the influential Ferguson model [15], which was instrumental in determining COVID-19 pandemic policy in the United Kingdom, is a SEIR model that divided society into 4 groups: S (“susceptible”), E (“exposed”; here, “E” refers to those who have been exposed, but who are not yet infectious [16]), I (“infectious”), and R (“recovered”). On the basis of these 4 dimensions, the dynamics of the pandemic can be described by modeling the transitions between groups. However, the social context is not modeled to generate a model that is not too complex and that can quickly generate rough approximations. However, there are also improved SEIR models that comodel, for example, quarantine status and intervention measures [17].

In addition to compartmental models, according to Gnanvi et al [18], statistical models have been used quite frequently during the COVID-19 pandemic. These are mostly growth models and time series models. Although statistical models often model one state (ie, one compartment at a certain point in time), statistical models, such as regression models, describe the statistical relationships between different independent variables and can provide information about the individual health status. Bayesian methods [19] and network models [20] and agent-based models [21] were also very elaborate but were used less frequently during the COVID-19 pandemic. No role during the pandemic played models based on artificial intelligence (AI) [22]. Finally, hybrid models that combine ≥2 of the presented approaches also exist. Looking at the application contexts of the individual models, it is striking that statistical and Bayesian models have been used to estimate epidemiological parameters, whereas compartmental models have normally been used to assess disease dynamics. Furthermore, simulation models that emerged during the COVID-19 pandemic found that the vast majority of models were SEIR models and statistical approaches [18]. This might change in the future as machine learning and AI models will become more prominent when computing capacities improve and new methodological approaches emerge. In addition, Eyert [23] hypothesized that agent-based modeling will become increasingly important in the context of scientific policy advice.

## Discussion

### Simulation Models as Visual Representations

What all these models have in common is that visual representations are often constructed from them. Visual representations of data not only give orientation in times of uncertainty but also frame the ways how we experience the pandemic. From the perspective of Latour [24], diagrams and statistics function as mediators that serve to make crises visible and tangible. In this sense, it can be argued that a crisis is created by its mappability as, for example, the “flatten the curve” line chart showed by illustrating how to slow down the pandemic to prevent an overstraining of health care services.

Although on the one hand, this imageability can be seen as an elementary tool of risk and crisis communication, there are also voices that consider visualizations as hidden normative claims, as through them certain world views and power relations can be produced and reproduced: “Visualizations are not neutral windows onto data; rather, they are the result of ‘judgement, discernment and choice’” [25]. In this respect, simulation models can only be interpreted as evidence-based tools for dealing with pandemics to a limited extent. In addition, this has to do with the fact that evidence in the case of simulation models cannot be measured empirically but rather follows plausibility-driven principles.

### Evidence-Based Models and Simulations in the Light of Ignorance and Normativity

As indicated earlier, evidence is often referred to in the context of certain practices of constructing and modeling uncertainty. It is true that quantitative modeling and the resulting number-based outcomes provide important bases for describing existential threats and generating political pressure for action. In contrast, precisely because of their numerical orientation, number-based recommendations run the risk of failing to account for possible bias effects [26]. These problems are related to the specific nature of modeling, which is often based on linear assumptions in the process of reducing complexity.

However, the principles by which complexity is reduced often remain opaque, although the results can be significantly affected by the model assumptions. Thus, when social and political complexity is translated into specific metrics and parameters, certain information inevitably falls by the wayside. Through the distinction between relevant and irrelevant data in relation to data collection and analysis, the notion of power comes into play in the context of data-driven technologies [27]. Consequently, the effectiveness and the legitimacy of nonlinear social and political dynamics are underestimated or even ignored. Such basic assumptions, based on modeling and simulation methodology, are also evident with respect to the pandemic, where policy responses were at times guided by calculations of relatively simple models that did not differentiate between specific social characteristics—a point of critique that might pave the way for a greater demand in agent-based modeling in the future.

In the end, what makes modeling a political phenomenon is less its calculative structure than the normative and analytical premises and biases, practices, and future visions in the social construction of this technology [28]. However, in some cases, neither the developers nor the users of technology seem to be aware that the social context contradicts the idea of a purely universal and instrumental character of technology [29]. In line with this perspective, social constructivist views, in contrast to theoretical perspectives that can be attributed to technological determinism, emphasize that science and technology are neither neutral nor objective, but the result of social processes that influence and structure the production of scientific knowledge as well as the innovation of technology [30-33]. In this view, technology can be seen as “normative social hardening” [34] because norms and values as an expression of certain cultural patterns become an inherent part of the construction of technology. If the construction process is to be seen as a materialized expression of ways of looking at the world, it is important to understand to what extent the normative orientations, attitudes, and premises that flow into the design of technology can be derived from culture- and context-specific circumstances, for example, how not only values and cultural meanings but also specific interests find expression in data-driven digital health technologies.

### Implications of Digital Health Simulations as Algorithmic Forms of Governance

To grasp the logic and implications of present and future pandemic technologies, it is helpful to explicate the approach of “algorithmic governance” [35] and to make it fruitful for the context of public health. The concept of “algorithmic governance” is examined primarily in its sociopolitical dimension to derive possible social and ethical implications. This is in line with the views of Hälterlein [12] that epidemiological computer simulations are to be understood as processes of algorithmic decision-making. In accordance with the 2 poles of technological determinism and social constructivism as presented earlier, different definitions of “algorithm” can be found. On the one hand, algorithms are assumed to be strictly rational affairs that combine the certainties of mathematics with the objectivity of technology [36]. In this perspective, the algorithm is understood as a “set of defined steps that, if followed in the correct order, will computationally process input (instructions and/or data) to produce a desired outcome” [37]. This definition follows an objectivist perspective that emphasizes the instrumental character of the algorithms. In this context, the formal correctness of the code is the only prerequisite to achieve desired effects. The existence of unintended side effects has been completely ignored.

In contrast, algorithms are conceptualized as “part of broader rationalities and ways of seeing the world” [38]. Roberge and Seyfert [39] shared this social constructivist-informed view. For them, algorithms are practical unfoldings and consequently are not simply reducible to code. Rather, they are to be understood as performative practices that involve a variety of rituals, narratives, and other symbolic forms of action [40]. Accordingly, algorithms are not to be understood as neutral, objective, or rational but as value laden and biased [41].

From another perspective, it follows that analytical approaches dealing with the design of algorithms must integrate all forms of social and material practices embedded in cultural, historical, and institutional contexts [42]. Hence, the assumptions behind algorithmic logic of sorting, filtering, searching, prioritizing, recommending, and deciding can be sufficiently illuminated [38]. These insights ultimately open the debate for a critical evaluation of algorithms, more specifically, the social meaning and contextual meanings of a code as “being contingent, ontogenetic, performative in nature” [43]. Only this kind of awareness of the contingency, heterogeneity, and diversity of practices “facilitates, rather than limits, critique, making our accounts more adequate to the practices they describe, and centering those practices as a site of dispute and potential regulation” [44]. According to Seaver [44], the critical analysis and regulation of algorithm-based governance presupposes that values, norms, and their sociocultural contexts can be considered and reflected on.

### The “End of Theory”

Following the view that agent-based modeling, as a subfield of computational social science, is becoming increasingly important regarding scientific policy advice, as exemplified in an impressive manner during the COVID-19 pandemic, the transition from probabilistic forms of uncertainty management to new forms of algorithmic prediction—which is particularly reflected in the rise of simulation models—is to be debated, especially because in the age of big data science, it is no longer based on testable hypotheses. This development radically points to an “end of theory” that manifests itself in a paradigmatic transformation of scientific work from causality to correlation, as algorithms find patterns that might remain hidden when classical scientific methods are applied: “Correlation supersedes causation, and science can advance even without coherent models, unified theories, or really any mechanistic explanation at all” [45]. In this respect, algorithms have lost their innocence in the age of big data.

This paradigm shift is also characteristic of the transition from the mode of probability to the mode of possibility with regard to future developments and potential threats. Thus, the logic of anticipation is complemented by another dimension that encompasses a variety of possible projected futures [46] as can be observed within the scenario-based approaches of pandemic simulation models. Here, objectivity is attributed to algorithms, although their effects often remain opaque [47]. The problem is that the establishment of this specific form of algorithmic governance that is based on obscure mathematical correlations promotes new dimensions of intransparency in several respects.

First, even with open-source codes, an understanding of how an algorithm works is reserved for special experts, especially in the case of correlative-associative procedures and AI applications, which will probably have greater significance in future pandemic management. Second, the black box of algorithmic governance is not a box that you only need to open to see the contents undisguised. Rather, it contains other black boxes [48] that have to do with the fact that code is not to be seen as a singular construction but rather as a network of assemblages that create the output. Third, this feature of nontransparency is further exacerbated when different algorithmic technologies are combined, becoming even larger “assemblages.” Following the perspective that those “assemblages are harder to be democratically controlled than more centralized forms of power” [34], a profound legitimacy problem arises.

### Algorithmic Governance as an Invisible Knowledge Regime

In particular, when the data-related selection and reduction process operates in terms of an opaque network structure, this marks a considerable loss of control and legitimacy, as it cannot be traced on which premises and normative assumptions decisions are made. Therefore, if algorithmic governance is understood as an invisible knowledge regime that produces interpretations of normality and deviation on the basis of digital data, which seep deeper into social processes and interactions and take on a life of their own, this speaks for the establishment of a subtle form of power whose legitimacy must remain largely unquestioned, because “The most profound technologies are those that disappear. They weave themselves into the fabric of everyday life until they are indistinguishable from it” [49]. In this perspective, algorithms appear not only as neutral sorting machines but also as performative instruments of domination that legitimize power.

Crucially, the performativity of algorithms [39] marks a change in that algorithms have left their original domain of mathematical problem-solving and instead increasingly shape and transform social life worlds. Against this background, it seems even more important to deal with ethical criteria, both in the context of development and the use of digital technologies, especially for public health. However, to comprehensively reflect on the ethical implications, it is necessary to understand the formulation of algorithmic procedures not as isolated expert knowledge but to analyze them in their social dimensions and references.

### Ethical Reflection Beyond Transparency and Acceptance

According to Amoore [50], algorithms and their social relations cannot be described simply by analyzing their code nor can ethics be coded into algorithms. Instead, Amoore [50] proposed to address the ethical responsibility of algorithms by identifying the sociotechnical conditions under which they emerge and function. To be able to grasp the ethicopolitical dimension of algorithms, it seems essential to confront the supposed unambiguity as an operational mode of algorithmic intrinsic logic with the multiplicity of social contexts. For Amoore [50], the error-proneness of algorithmic predictions results not from formal technical-mathematical inadequacies but from the fundamental contingency and uncertainty of human existence: “The madness of algorithms does not lie in the moral failure of the designer or the statistician but is an expression of the forms of unreason folded into a calculative rationality that reduces the multiplicity of potentials to a single output.” Consequently, it cannot be a matter of making algorithmic procedures transparent to gain legitimacy.

However, the invisibility and unaccountability of algorithmic power imply that the focus on the acceptance of algorithmic governance technologies does not seem to be sufficient to address questions of legitimacy as this is undermined both by normalization effects and by performativity. Instead, it is necessary to explicitly consider questions of acceptability. Although social acceptance refers to the fact that new technology is accepted or merely tolerated by a community, ethical acceptability refers to a conceptual reflection of the technology that takes into account the moral issues that emerge from the introduction of new technologies. In this way, for example, the contradiction that risky technology is accepted for morally wrong reasons can be critically reflected upon, which would be lost if the focus were solely on acceptance within purely empirically oriented research approaches [51]. Consequently, an ethical perspective must take special account of the nonquantifiable aspects of human life not least because this is also where the key to trust in technological applications lies [52].

### Political Implications of Algorithmic Public Health Simulations

In the following section, I relate the critical perspective on algorithmic governance presented to the application context of public health simulations to shed light on the implications of mathematical modeling. This highlights the simulation of pandemic crises and thus the question of how public health management is changing by the treatment of emerging infectious diseases through simulation.

As illustrated above, although numbers “per se do not claim neutrality, truth, or scientific authority, they contribute to create realities, communities, policies and public concern” [53]. This implies, for example, that policy makers can select specific evidence to either support or prevent a lockdown [54]. From this point of view, public health simulations have the potential to contribute to the stabilization of power positions of political decision makers. In this respect, “images of epidemics and zoonoses are not mere representations of infectious diseases and their social impact, but rather actants in a broader political economic arena of power and knowledge” [55]. Consequently, the use of simulation models enables responsible emergency actors to perform within the regime of public health by referring to their capacities to govern rationally [56].

Among other things, the issue of power hierarchies raises questions about the role and functions of policy advice in times of health crises and directs attention to the discursive significance of certain forms of knowledge in relation to policy decisions. It is thus necessary to clarify whether the evidence—however, this is to be determined—is sufficient as a guide for political decisions or whether there is a danger of an “epistemization of the political” [57] if politics simply follows scientific claims to validity. Ultimately, this also raises the question of the principles on which political decisions should be based on value-based trade-offs or on the “best” scientific knowledge. As politics as an extended arm of scientific findings would practically render itself superfluous and because a hierarchization of scientific findings cannot easily be made anyway, there is much to be said for the idea that politics should not be knowledge based but value based. However, despite this insight, there were repeated voices during the pandemic arguing for a stronger scientific foundation for political decisions.

Against this background, it is important to ask what kind of scientific knowledge appears relevant. The political reactions at the beginning of the pandemic, for example, were largely characterized by a mobilization of medical and epidemiological knowledge that formed the basis for the creation of simulation models.

However, if one interprets the COVID-19 pandemic not only as a challenge in the sense of public health but also as a social crisis, many arguments can be found for considering social science knowledge in the context of more interdisciplinary expert panels and general crisis response modes. In this regard, crisis responses could benefit from an expansion of the epistemic corridors beyond natural science knowledge production [58].

When “evidence” is not necessarily unambiguous and it seems possible that forecasts deviate greatly from “reality,” performative dynamics [59] can become a problem. To capture the sociotechnical dynamics of algorithmically structured public health technologies, we need an ethical perspective that goes beyond the analysis of individual acceptability and explicitly problematizes questions of social acceptability. In doing so, ethical reflection must not only refer to criteria, such as transparency or legitimacy but must, according to Amoore [50], rather keep an eye on the interrelations between society and technology to be able to unfold a holistic critical perspective.

A central question then would be which political options are and were represented in pandemic simulation models. What has not been modeled is at least as interesting. Eyert [60], for example, was able to show with regard to Germany that elementary influencing variables, such as leaving schools and daycare centers open instead of closing them down, were not integrated into any of the models. Hence, if certain political courses of action are not represented in the simulations, it highlights the power that arises from a possible selectivity of the modeled parameters. Particularly regarding the problem of accountability in algorithmic governance technologies, this question becomes more urgent when political decisions no longer appear to be attributable, and a substantial democratic deficit is derived from this with a high degree of diffusion of political responsibility.

Ethical reflection on pandemic simulations must therefore not only address the problem that evidence hardly existed at the beginning of the COVID-19 pandemic and the role of ignorance in scientific advice was too short. Rather, it must be actively reflected and debated whether what McGeoy [61] describes as “strategic ignorance” can be observed in relation to political decisions and the corresponding epistemic attempts at legitimation. In this respect, the discrimination potentials of pandemic simulations must be considered seriously if, for example, social inequalities in handling health crises are ignored. This manifests in sociostructural indicators: underprivileged status groups such as poor or health-impaired people, single parents, and cramped living conditions.

To adequately analyze the “counterproductive effects of technologies” [62] in the context of public health crises from a critical ethical perspective, a comprehensive consideration of the social construction principles and implicit premises as well as the unintended or unexpected side effects, for example with regard to the performative and temporal dynamics of implementation in the use context, of mathematical modeling is necessary. This touches not only on issues of transparency and possible discrimination but also on the problem that social complexity cannot simply be translated into binary algorithmic codes without risking significant information loss. Only a holistic ethical reflection, which is situated on several levels and illuminates the criteria of social acceptability besides individual acceptability, can take into account the complexity of implications in the use of pandemic simulation models.

### Operationalized Overview on Key Issues of Pandemic Crisis Technologies

Textbox 1 presents an operationalized overview of all key aspects and reflections addressed in this contribution. It also contains 3 major recommendations that might be relevant to present and future public health technologies.

**COVID-19 pandemic simulations for public health management: social construction and the implications.**

**Social construction of public health technologies**

- What is evidence?
  Technology is never neutral: charts and curves as quasiobjective representations of a questionable neutrality of scientific knowledge
  Manifest and latent assumptions, values, and norms affect data collection, analysis, and interpretation
  Social complexity cannot be transferred easily into binary code structures
- Paradoxes and ambivalences of knowledge
  Paradoxes, eg, ignorance within pandemics versus rise of digital health technologies
  Ambivalences of knowledge: new knowledge creates new ignorance
- Algorithmic governance and participation
  Technology versus participation: find a balance between algorithmic governance and other forms of coping with uncertainties and crises (eg, social participation)
  Technology and participation: participatory design as a mode of innovating public health technologies

**Social, ethical, and political implications of public health technologies**

- Social implications
  Entanglements between knowledge and power, eg, within pandemic images and charts
  Accountability problems and unintended side effects of digital health technologies
  Performativity, eg, with regard to the mappability of a pandemic in the context of political power relations
- Ethical implications
  Acceptance versus acceptability: distinguish between use attitudes and ethical criteria
  Acceptability affects legitimacy and trust
  Transparency and legitimacy not necessarily directly correlated (argument by Amoore [50])
- Political implications
  Strategic ignorance: What scenario is modeled? But also: What is not modeled?
  Science-policy nexus: “epistemization of the political”
  Danger of control illusions if political decisions are merely data-based

The following were the three main recommendations:

1. Consider health crises also as social and political crises.

2. Merge crisis knowledge within interdisciplinary forms of pluralistic knowledge production: socioscientific knowledge and social participation as precious resources in reacting to health crises.

3. Be aware of the connectedness of the social construction principles and the various implications of public health technologies (especially regarding algorithmically driven practices and tools).

One key question was how the use of digital data is changing the way governments address ethical and societal questions in public health crises. Simulations were reconstructed as visual representations and sources for legitimate political power constellations. In addition, the principles of mathematical modeling based on algorithmic command structures were determined as an intransparent mode of dealing with crises and uncertainties, which relate less to individual acceptability than to the level of acceptability. To judge the acceptability of mathematical or algorithmic modeling techniques as an ethical reflection of technologies, we must shed light on the premises, values, and norms in the social construction process of generating such models and simulations, for instance, by communicating and reflecting the assumptions. For this, it is also crucial to reflect on the role of ignorance as a problem of technocratic, data-driven crisis governance technologies.

In addition, it has been argued that alternative ways of knowledge production in times of health crises should be identified. In the context of the often-diagnosed lack of data, for example, in relation to social inequalities during the pandemic, greater importance of social science knowledge in pandemic crisis responses could be a useful and necessary complement to purely medical and epidemiological strategies for dealing with public health crises. The role of interdisciplinary work in the development and implementation of digital medical applications could also be enriched by participatory methods of technological innovation to maintain trust in technological public health solutions.

To take the criticism of technological solutionism seriously, social and more experimental forms of dealing with crises could also be debated and tested. Only in this manner can public health management escape the accusation of a technology-based top-down strategy that lacks democratic legitimacy. Considering a global preparedness regime that is able to detect public health threats at an early stage, an expansion of the technological architecture in the form of a “positive biopolitics” [63] to establish adequate structures of intervention on a planetary scale at an early stage, which should enable a higher degree of inclusion, care, and prevention, seems worthy of discussion. Against this background, this paper should not be understood as a plea against the use of technologies to address public health crises. Rather, it is a proposal to relate different forms of knowledge to each other in an interdisciplinary approach in such a way that they make the best possible use of the potentials of the heterogeneity inscribed in them and at the same time proactively consider ethical criteria of acceptability.

### Limitations

Overall, it is difficult to determine the direction in which the development and implementation of pandemic crisis technologies will evolve. Therefore, it is also difficult to estimate which concrete application areas can be found for agent-based models or AI-based approaches within future simulation models, for example, and to what extent these will raise completely different implications from those outlined. New and surprising issues and challenges could soon emerge, especially as public health technologies in the aftermath of the COVID-19 pandemic represent a rapidly developing field.

### Conclusions

Pandemic simulation models are an important tool to support the necessary political decision-making in crisis situations. However, their informative value not only depends strongly on the quality of the available data but also, at the same time, raises diverse implications on different levels of concern. The modes of reducing complexity within simulation models are essential, as is the question of how data quality can be optimized in the first place with regard to the modeling of social complexity, which tends to increase further. Although AI failed to exhibit its potential during the pandemic, with regard to simulations, there are indications that AI models will sooner or later become more important in the context of public health management. Thus, the ambivalences of simulation models will probably continue to be the subject of ethical reflections and sociopolitical issues in the future.

## Abbreviations

AI: artificial intelligence
SEIR: susceptible, exposed, infectious, recovered
SIR: susceptible, infectious, recovered
